# Association between metabolic syndrome components and the risk of developing nephrolithiasis: A systematic review and bayesian meta-analysis

**DOI:** 10.12688/f1000research.28346.1

**Published:** 2021-02-11

**Authors:** Ilham Akbar Rahman, Ilham Fauzan Nusaly, Syakri Syahrir, Harry Nusaly, Makbul Aman Mansyur

**Affiliations:** 1 Faculty of Medicine, Hasanuddin University, Makassar, South Sulawesi, 90222, Indonesia; 2 Department of Urology, Faculty of Medicine, Hasanuddin University, Makassar, South Sulawesi, 90245, Indonesia; 3 Department of Internal Medicine, Faculty of Medicine, Hasanuddin University, Makassar, South Sulawesi, 90245, Indonesia

**Keywords:** Metabolic syndrome, Hypertension, Obesity, Dyslipidemia, Diabetes, Nephrolithiasis

## Abstract

**Background:** There is increasing evidence that nephrolithiasis is a systemic disease, as opposed to an isolated urinary metabolic problem, after considerable links were found between nephrolithiasis and systemic diseases such as hypertension, obesity, dyslipidemia, and insulin resistance. The interplay between these four factors defines metabolic syndrome (MetS). In this review we aim to clarify the associations of MetS and its components to kidney stone incident.

**Methods: **Online databases of EMBASE, MEDLINE, and Google Scholar were searched from January 1998 up to October 2020 to identify observational studies examining the association between metabolic syndrome components and kidney stone incident. Bayesian random-effects meta-analysis and meta-regression were performed to observe the association. Linear dose-response analysis was conducted to shape the direction of the association. Data analysis was performed using STATA, and R statistics.

**Results:** A total of 25 potentially relevant studies (n = 934,588 participants) were eventually identified. The pooled results suggested that metabolic syndrome was associated with an increased risk of nephrolithiasis with an odds ratio (OR) of 1.769 (95% CI: 1.386 – 2.309).  The summary OR of hypertension and dyslipidemia for developing nephrolithiasis were 1.613 (95% CI: 1.213 – 2.169) and 1.586 (95% CI: 1.007 – 2.502) respectively. The presence of diabetes mellitus and obesity had an OR of 1.552 (95% CI: 1.027 – 2.344) and 1.531 (95% CI: 1.099 – 2.109) respectively. Our results revealed that the increasing number of MetS traits will increase the risk of developing nephrolithiasis, the higher the fasting plasma glucose, and body mass index, the higher the risk of kidney stones incident.

**Conclusions:** Our results suggest that hypertension, diabetes, obesity and dyslipidemia are associated with increased risk of developing nephrolithiasis. Linear significant association between MetS components and nephrolithiasis were revealed in our study which reinforced the notion that should be considered a systemic disorder.

## Introduction

Kidney stone disorder, characterized by abnormal urine composition and dehydration, is an increasingly common condition that is most common in older men ( Shoag *et al.*, 2015). Its incidence and occurrence has grown progressively and globally for decades. It has been reported that nephrolithiasis was found in all ages, peaking between the ages of 20 and 60 where it rapidly increases between the ages of 40 to 59 ( Hiatt *et al.*, 1982).

In recent years, nephrolithiasis is hypothesized to be a systemic disorder which requires more attention and evaluation in primary health care. The prevalence of kidney stones has increased and been associated with the components of metabolic syndrome including obesity, elevated blood pressure, dyslipidemia, and glucose intolerance ( Obligado & Goldfarb, 2008; Sakhaee, 2008; Sakhaee, 2009; Sakhaee & Maalouf, 2008). As opposed to an isolated urinary metabolic problem, research showed that it was related to an increased risk of several comorbidities such as coronary heart disease, stroke, renal cell carcinoma and end-stage renal disease (ESRD) ( Dhondup *et al.*, 2018; Peng & Zheng, 2017; van de Pol *et al.*, 2019). In addition to being a prevalent and costly problem, recent evidence also showed that urinary stones are associated with several metabolic traits such as hypertension, diabetes mellitus (DM), and obesity ( Kovshilovskaya *et al.*, 2012; West *et al.*, 2008). Comprehensive evidence shows that the association seems to be reciprocal: either stone formers seem to induce MetS or ones with MetS induce the increasing risk of kidney stones ( Reiner *et al.*, 2011). Several meta-analyses have found an association between hypertension and diabetes mellitus in nephrolithiasis patients ( Aune *et al.*, 2018; Shang *et al.*, 2017). However, there are no previous studies that have compared all possible metabolic syndrome traits in determining which traits have the most significant influence for the risk of nephrolithiasis and to determine the direction of the association. Moreover, due to sample size problems individual studies may not have enough - statistical power, therefore we performed a systematic review and meta-analysis to determine the association between all possible metabolic syndrome traits to assess the most significant risk for developing nephrolithiasis from all the evidence. Meta-regression and dose response analysis were performed to assess the direction of association. All these analyses were implemented in a Bayesian framework so that we could provide the results with more confidence ( O’Hagan, 2004; Spiegelhalter, 2004). Our study outcome could clarify the association between metabolic syndrome and its components to the risk of developing nephrolithiasis.

## Methods

### Literature search strategy and literature selection

Relevant articles were retrieved from online databases including EMBASE, MEDLINE and Google scholar from January 1998 until October 2020. The search strategy consisted of two parts (metabolic syndrome and nephrolithiasis) using specific keywords combined with medical subject heading (MeSH) terms and words: "metabolic syndrome ", "hypertension", "diabetes mellitus", "insulin resistance", "dyslipidemia", "obesity", “waist circumferences”, “hypertriacylglycerolemia”, “hypertriglyceridemia”, “kidney stones”, “nephrolithiasis". The full texts and abstracts were originally and independently selected by two reviewers. Full texts and abstracts were scored according to the inclusion and exclusion criteria. The studies which did not fit with the inclusion criteria were excluded. Discrepancies between the two reviewers were resolved through a discussion with a third reviewer. Ethical approval was not required because the data did not contain individual patient information.

### Data extraction and quality assessment

**Figure 1.**
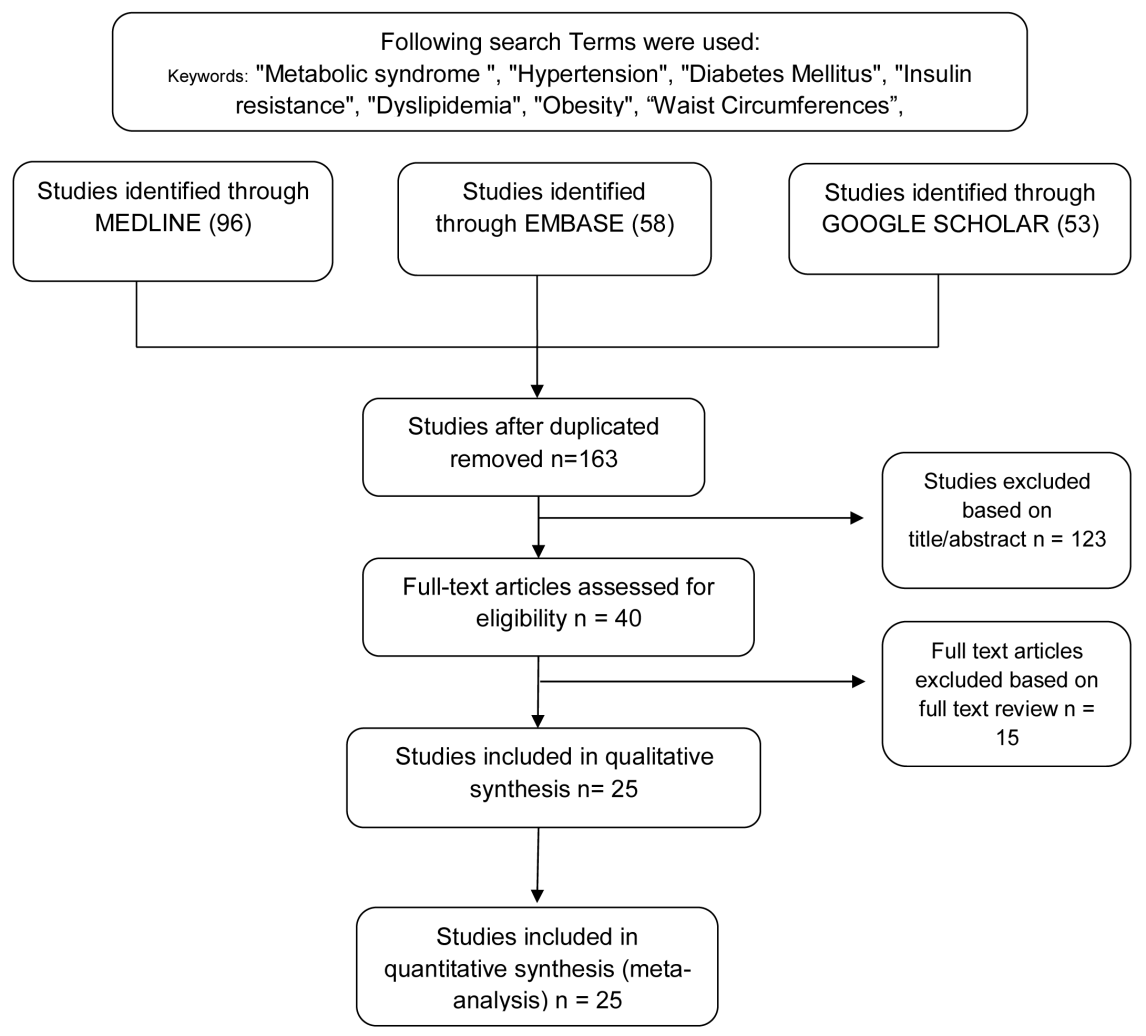
Flowchart of systematic literature identification and selection process implementing Preferred Reporting Items for Systematic Reviews and Meta-Analyses guide.

The studies included in this article met the following criteria: (1) the subjects were nephrolithiasis patients; (2) research was conducted as observational study to show causal-effect relationship (case control or cohort studies); (3) the study showed association as risk with 95% CI; (4) the language of the article was published in English. Two reviewers (IAR and IF) separately extracted and analyzed the data based on study selection criteria using standardized, structured and pilot extraction forms. The results were reviewed and discussed by IAR and IF to complete the included studies. Any discrepancies were resolved in a discussion with a third reviewer. For each included study, several pieces of information were extracted including author name, year of publication, number of sample sizes, mean age, study design, countries, number of participants and cases, study period, predictors and outcomes. Table 1 comprehensively shows all of the above data. Newcastle Ottawa Scale (NOS) tool was used to assess the relevance of the included studies and the strength of the evidence. Figure 1 shows the detailed literature search and selection process as PRISMA guideline was followed. Publication bias was investigated by the Begg and Egger tests.

**Table 1.** Baseline characteristics of included studies.

Author, year	Metaboli Syndrome Traits	Study design	Mean Age	Country	Study Population
**Sandra L. Ramsey, 2004**	**Hypertension**	**Cross-sectional** **study**	**37**	**US**	**2818**
**Francois Madore, 1998**	**Hypertension**	**Prospective cohort** **study**	**54**	**US**	**47418**
**Yen-Tze Liu, 2017**	**Hypertension, Low HDL, Obesity,** **Metabolic Syndrome**	**Cross-sectional** **study**	**46**	**Taiwan**	**3886**
**Francesco P. Cappuccio, 1999**	**Hypertension**	**Prospective cohort** **study**	**46.5**	**Italy**	**503**
**Massimmo Cirillo, 1988**	**Hypertension**	**Cross-sectional** **study**	**NA**	**Italy**	**5376**
**Sheng-Han Tsai, 2018**	**Hypertension, Obesity, DM, Dislipidemia**	**Retrospective cohort study**	**43.3**	**Taiwan**	**39124**
**In Gab Jeong, 2011**	**Hypertension, Obesity, DM,** **Low HDL, Metabolic Syndrome**	**Cohort** **retrospective**	**50**	**Korea**	**34895**
**Hao Ping, 2019**	**Hypertension, Obesity, DM**	**Cohort prospective**	**46.23**	**China**	**9667**
**Xiang Shu, 2017**	**Hypertension, Obesity**	**Cohort prospective**	**52**	**China**	**127220**
**Loris Borghi, 1999**	**Hypertension**	**Cohort prospective**	**42**	**Italy**	**267**
**Saloua Akoudad, 2010**	**Hypertension**	**Cohort prospective**	**60**	**US**	**12161**
**Elichi Yoshimura, 2015**	**Obesity**	**Cohort prospective**	**31**	**Japan**	**4074**
**Eiji Oda, 2014**	**Obesity**	**Cohort retrospective**	**51**	**Japan**	**2718**
**Mathew D. Sorensen, 2014**	**Obesity**	**Cohort prospective**	**63.5**	**US**	**84225**
**Eric N. Taylor, 2005**	**Diabetes Mellitus**	**Cohort prospective**	**47**	**US**	**220478**
**Mu Tsun Shih, 2016**	**Hypertension, Diabetes Mellitus,** **Dyslipidemia**	**Cohort retrospective**	**44**	**Taiwan**	**3344**
**James H. Masterson, 2015**	**Diabetes Mellitus, Dyslipidemia**	**Cohort retrospective**	**31**	**US**	**52184**
**Qi Ding, 2018**	**Dyslipidemia**	**Case control study**	**55**	**China**	**1196**
**Domenico Rendina, 2009**	**Low HDL**	**Cohort prospective**	**63**	**Italy**	**2132**
**Ho Won Kang, 2014**	**Low HDL**	**Cohort retrospective**	**47**	**Korea**	**2620**
**In Ho Chang, 2011**	**Metabolic Syndrome**	**Cohort prospective**	**42**	**Korea**	**3872**
**Yang-Ju Kim, 2013**	**Metabolic Syndrome**	**Cohort retrospective**	**45**	**Korea**	**116536**
**Yasuo Kohjimoto, 2013**	**Metabolic Syndrome**	**Cross-sectional** **study**	**52**	**Japan**	**11555**
**Bradford West, 2008**	**Metabolic Syndrome**	**Cross-sectional** **study**	**52**	**US**	**14870**
**A. J. Landgren, 2017**	**Hypertension, Obesity**	**Cohort prospective**	**69**	**Sweden**	**131449**

### Outcome

Primary endpoint was the occurrence of kidney stones with the predictors assessed as metabolic syndrome traits. Secondary endpoint was the direction of association assessing between the dose of the predictors and the response of kidney stones occurrence.

### Statistical analysis

We performed an empirical Bayesian random effect meta-analysis and meta regression to understand the association between predictors and outcome of kidney stones. All data were analyzed using R language (version 1.2.1335), STATA (version 12.0; Stata Corp LP, College Station, TX) and R. An odds ratio (OR) with a 95% confidence interval (CI) used for each association was generated using Bayesian meta-analysis. The comparison of risk factors with each other was conducted based on OR, where HRs (Hazard Ratio) and RRs (Risk Ratio) were both directly considered as OR ( Greenland, 1987), for kidney stones outcome by performing the simulation with 16,000 iterations and with the burned out of 10,000 iterations in the Bayesian meta-analysis model. Chi-squared based on Q-statistic test (P<0.10) and I ^2^ statistic quantification were used to assess heterogeneity of studies. I ^2 ^values of heterogeneity were interpreted as insignificant (0–25%), low (26–50%), moderate (51–75%) and high (>75%) ( Higgins *et al.*, 2003). Empirical Bayesian random effects meta-regression was further conducted to explore the impact of moderators in the study effect size. Linear dose response analysis was conducted to shape the direction of association. Sensitivity analyses were conducted to explore various levels of heterogeneity between studies and observe how the results will change for different values of between study variance τ ^2^ and the heterogeneity statistic *I ^2^*. Publication bias and small study effect were evaluated by funnel plots, Egger’s regression test and Begg’s rank correlation test.

## Results

### Search results and included strategies

This meta-analysis study included a total of 207 studies found using the search strategy. A full text review and abstract selection were then performed and 138 studies were excluded due to reviews, case reports, duplicates, and not in English. Finally, 25 studies met the inclusion criteria of 934,588 patients, which were eligible and added for further analysis. A block diagram of the procedure for searching and selecting studies is shown in Figure 1.

### Relationships between metabolic syndrome and nephrolithiasis

**Figure 2.**
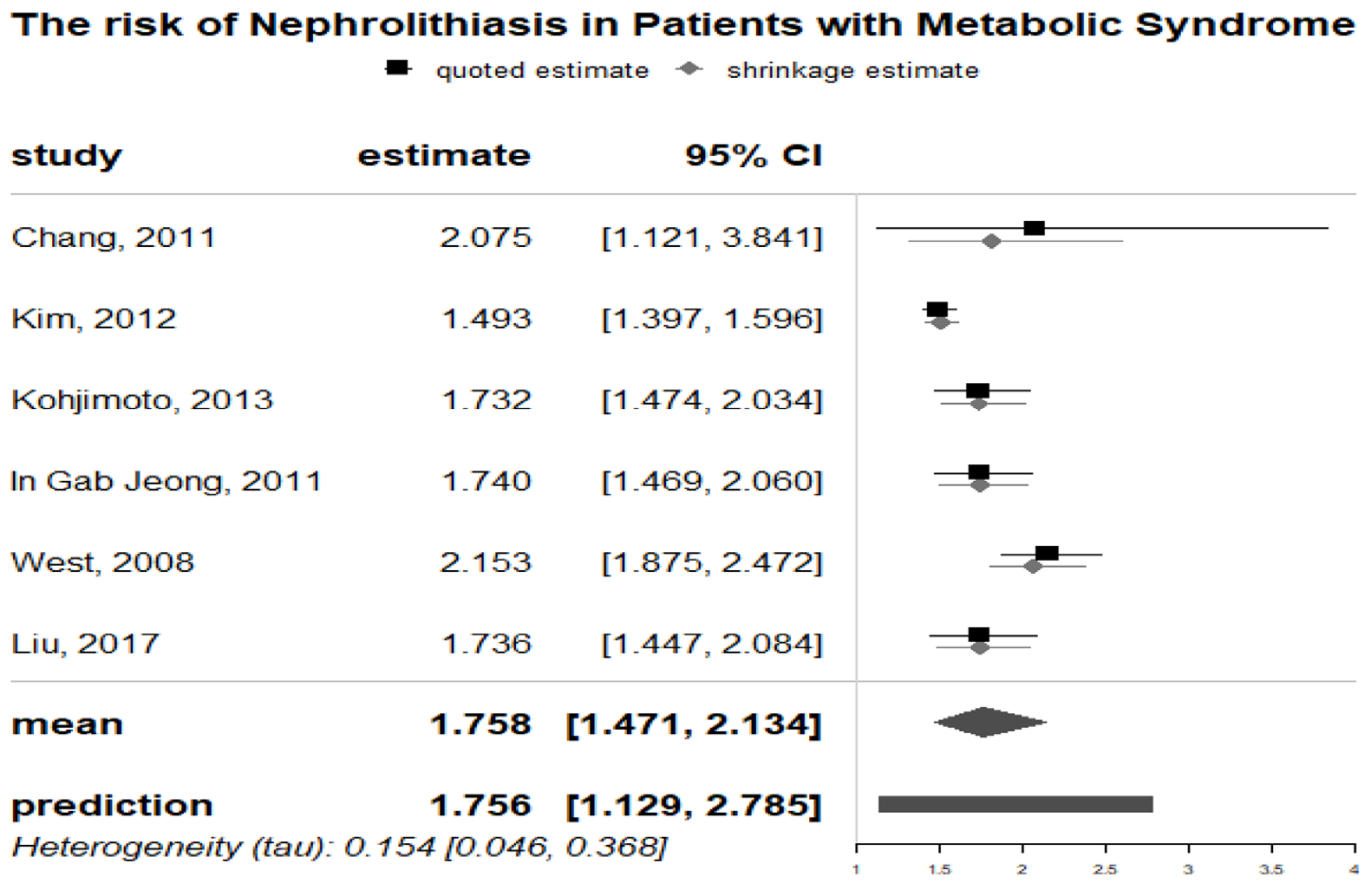
Forrest plot of nephrolithiasis risk in metabolic syndrome patients.

As demonstrated in Figure 2, prior metabolic syndrome was significantly associated with the mean risk of nephrolithiasis (OR: 1.769; 95% CI, 1.386-2.309) in the meta-analysis of 6 studies. There was low heterogeneity (Q test; I2 = 66.84%). Also, publication bias and small study effect were not found based on the symmetry of the funnel plot (Supplementary material 1a ( Rahman *et al.*, 2021b)) and the Begg’s rank correlation test (P = 0.469) and Egger’s regression test (P= 0.3093) tests. The pooled ORs remained significant after 3 more studies were added to both meta-analyses using the trim-and-fill method (Supplementary material 1b ( Rahman *et al.*, 2021b)). Sensitivity analyses were consistent for different values of study variance and heterogeneity (Supplementary material 1c ( Rahman *et al.*, 2021b)).

### Relationships between hypertension and nephrolithiasis

**Figure 3.**
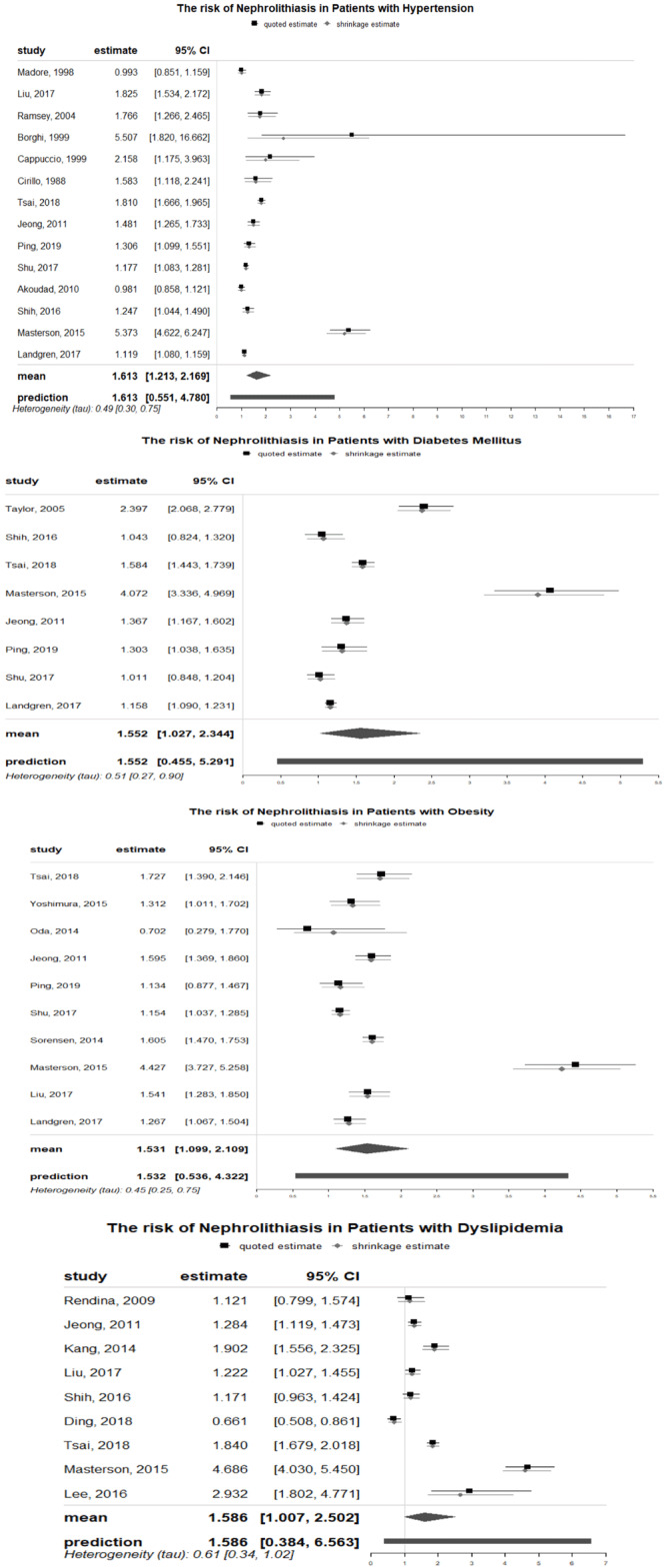
The association of metabolic syndrome traits and kidney stones incident using empirical Bayesian meta-analysis.

As demonstrated in Figure 3, prior HTN (hypertension) was significantly associated with the risk of nephrolithiasis (RR: 1.613; 95% CI, 1.213-2.169) in the meta-analysis of 14 observational studies. There was high heterogeneity (Q test, P < 0.001; I 2 = 98%). Also, publication bias and small study effect were not suggested based on the symmetry of the funnel plot and the Begg’s (P = 0.233) and Egger’s (P= 0.1386) tests (Supplementary material 2a ( Rahman *et al.*, 2021b)). The pooled ORs remained significant after 6 more studies were added to both meta-analyses using the trim-and-fill method (Supplementary material 2b ( Rahman *et al.*, 2021b)). Sensitivity analyses were consistent for different values of study variance and heterogeneity. (Supplementary material 2c ( Rahman *et al.*, 2021b)).

### Relationships between DM and nephrolithiasis

The results of meta-analyses that assessed the relationship between diabetes and nephrolithiasis were shown in Figure 3. DM was significantly linked to increased risk of nephrolithiasis formation (RR: 1.552; 95% CI, 1.027-2.344). For incident nephrolithiasis with Begg’s (P= 1.000) and Egger’s (P = 0.3106) tests revealed no publication bias however funnel plots were asymmetrical with a few dots outside the intervals (Supplementary material 3a ( Rahman *et al.*, 2021b)). There were no new studies added by the trim-and-fill method and the results of sensitivity analyses remained consistent in different values of study variances and heterogeneity (Supplementary Item 3b and 3c ( Rahman *et al.*, 2021b)). High heterogeneity existed in the analyses of nephrolithiasis incident (Q test, I ^2^ = 97%)

### Relationship between obesity and nephrolithiasis

The pooled analysis of OR of 10 individual studies showed that patients with obesity had overall RR to the risk of nephrolithiasis of 1.531 [95%CI: 1.099–2.109]. Publication bias and small study effect were not found based on symmetry of the funnel plot (Supplementary material 4a ( Rahman *et al.*, 2021b)) and the results of Begg’s (P = 0.8618) and Egger’s (P = 0.9990) test. After four more studies added to meta analyses by using the trim-and-fill method, the pooled ORs remained significant (Supplementary material 4b ( Rahman *et al.*, 2021b)). Despite high heterogeneity existed (Q test, I ^2^ = 96%) between studies, the results of sensitivity analyses remained consistent in different values of study variances (Supplementary material 4c ( Rahman *et al.*, 2021b)).

### Relationships between dyslipidemia and nephrolithiasis

There were significant associations between dyslipidemia and nephrolithiasis as demonstrated in Figure 3. Specifically, patients with dyslipidemia were 1.586 times more likely to develop nephrolithiasis (95% CI, 1.007 -2.502). In this pooled analysis, the heterogeneity was substantial with I ^2^ = 97%. The funnel plot of studies assessing dyslipidemia and the risk of nephrolithiasis indicated potential publication bias that was however not supported by the result of Begg’s (P = 0.9195) and Egger’s (P = 0.5712) tests (Supplementary Item 5a ( Rahman *et al.*, 2021b)). The pooled ORs remained significant after 1 new study added with the trim and fill analysis. Sensitivity analyses yielded consistent results (Supplementary Item 5b and 5c ( Rahman *et al.*, 2021b)).

### Meta regression and dose response analysis

**Figure 4.**
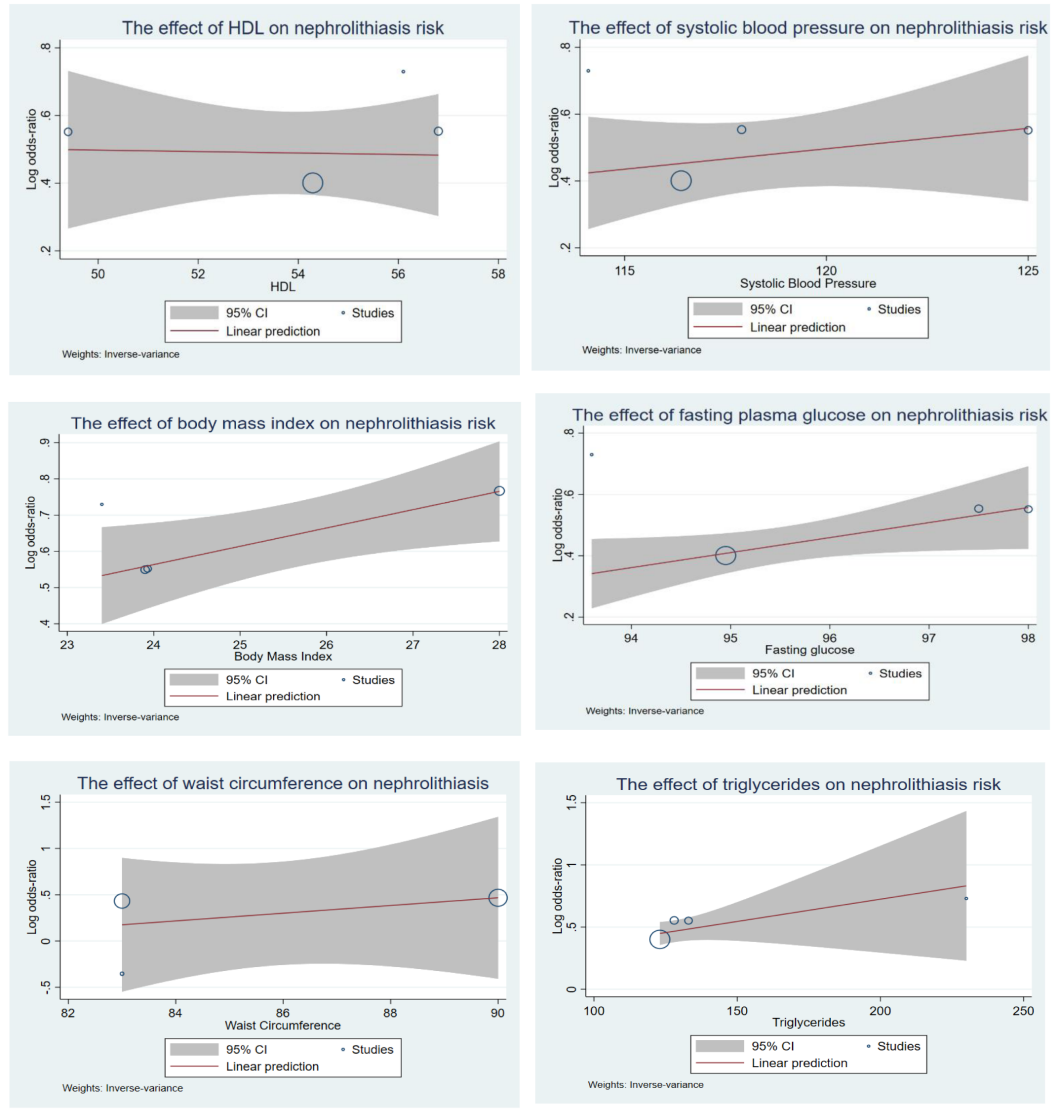
The association of metabolic syndrome traits and risk of kidney stones incident using Empirical Bayesian meta regression.

**Figure 5.**
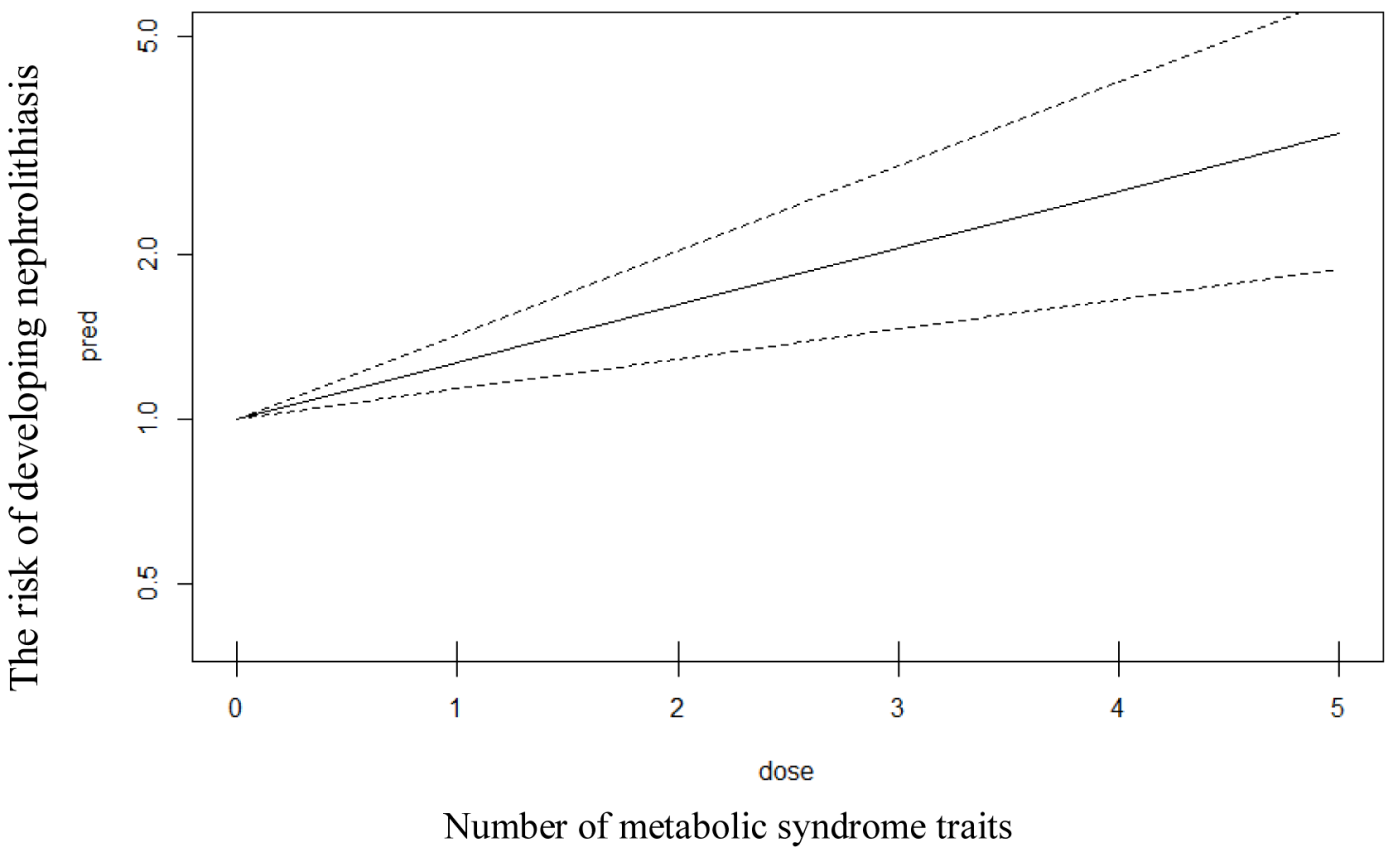
Linear model of dose response analysis between number of metabolic syndrome traits and the risk of developing nephrolithiasis.

As demonstrated in Figure 4, the effect of metabolic syndrome traits on kidney stone formation was also assessed by using Bayesian meta regression in six reports on the study of MetS and nephrolithiasis. Stratification for hypertension, dyslipidemia, obesity, and diabetes were conducted. Moreover, linear dose response analysis was also conducted as shown in Figure 5. Our analyses revealed that the incident of nephrolithiasis was significantly associated (p value <0.05) with the increasing of age. Fasting plasma glucose and body mass index were also reported to significantly associated (p value <0.05) with incidence of kidney stones. The more increased the fasting glucose and body mass index then the more increased the risk of kidney stones incident. Linear correlation was also found in metabolic traits where the increasing number of MetS traits will increase the risk of developing nephrolithiasis. For the effect of blood pressure, there is a trend that the more increased blood pressure then the more increased risk of kidney stones, however the result was not significant (p value > 0.05). HDL (high density lipoprotein) level revealed no trend and significance (p value > 0.05) for the association with kidney stone incident. 

## Discussion

To the best of our knowledge, this is the first systematic review study to confirm the associations between nephrolithiasis and metabolic syndrome components using Bayesian meta-analysis with meta regression and dose response analysis. All studies selected for meta-analysis were of high quality with NOS ≥7. Our results indicated significant relationships between kidney stones and metabolic syndrome traits such as hypertension, diabetes, obesity, and dyslipidemia. Although heterogeneity existed across most analyses, the results were consistent in all sensitivity analyses and trim-and-fill tests, suggesting these relationships were relatively robust and unlikely to be the consequence of confounding.

Our results indicate that hypertension has been associated as a risk factor in the development of nephrolithiasis. Our result is also in coherent with ( Cappuccio *et al.*, 1990) where we found that the risk of kidney stones development was increased stepwise with the severity of hypertension that the higher the blood pressure, the higher the risk in developing nephrolithiasis. The relationship could be clarified by many possible mechanisms. Hypercalciuria or increased renal calcium excretion was identified as the first theory concerning the relation between hypertension and nephrolithiasis. This hypothesis was also supported in previous studies that HTN has increased the occurrence of kidney stones, by its association with hypercalciuria, hyperoxaluria, and hypocitraturia ( Borghi *et al.*, 1999; Coe *et al.*, 2010). This hypothesis was strengthened by research ( Coe & Bushinsky, 1984; Pak *et al.*, 1975; Resnick *et al.*, 1986) showing that hypertensive and stone-forming patients often have certain hormonal correlations, such as elevated levels of 1,25(OH)2D and parathyroid hormone, which may justify the finding of hypercalciuria in large subgroups of both subjects. This hypothesis is linked for stones containing calcium, including calcium oxalate and calcium phosphate ( Coe *et al.*, 2010). A primary renal tubular defect (the 'renal calcium leak' hypothesis) or the result of central volume expansion observed in hypertension (the 'central blood volume' hypothesis) may be attributed to increased urinary calcium excretion. This disorder is well evident in the Italian cohort studied by Borghi *et al.* ( Borghi *et al.*, 1999), where urinary calcium excretion was higher in hypertensive patients, both men and women, than in normotensive patients. Previous authors also advance the hypothesis that a pathogenic relation between hypertension and nephrolithiasis may be caused by the increased renal calcium excretion ( Devynck *et al.*, 1984; McCarron, 1985; Resnick, 1987). Previous research also revealed that there is an existence of calcium metabolism alterations, including increased renal excretion both in hypertensive animals ( Hsu *et al.*, 1986; Wexler & McMurtry, 1981) and humans ( Gennari *et al.*, 1986; Strazzullo *et al.*, 1983). Furthermore, it is understood that these conditions are aggravated by a high dietary intake of sodium chloride. High dietary salt intake will in particular, facilitate hypertension by volume expansion and stone forming through increasing urinary calcium excretion and reducing citrate ( Canessa *et al.*, 1980; Postnov *et al.*, 1979). This finding was also confirmed by research ( Borghi *et al.*, 1999) showing that in many hypertensive patients, the effect of NaCl intake appears to be important in inducing high blood pressure and causing high calcium excretion. It is well known that a small increase in urinary oxalate increases the lithogenic risk of calcium oxalate, while hyperuricosuria can encourage both uric oxalate production ( Robertson & Peacock, 1980). Genetic theory also describes how genetics is associated with calcium metabolism alterations in hypertension and kidney stone incident. A previous study ( Cirillo *et al.*, 1989) also reported that alteration in renal calcium handling in hypertensive rats led to higher urinary calcium excretion. Other epidemiological studies tend to indicate that hypertensive subjects and stone formers have common dietary patterns, distinguished above all by a low consumption of calcium ( McCarron & Morris, 1987; McCarron *et al.*, 1984; Menon, 1993), which, as is well known, can contribute to increased oxaluria due to increased intestinal oxalate absorption. This theory is born out of the fact that the reduction of arterial pressure levels and reduction of the likelihood of renal stone development appear to be supported by calcium-rich diet ( Curhan *et al.*, 1997).

A significant association of DM with the risk of nephrolithiasis was reported in our research. It is well known that by deranging ammoniagenesis and increasing sodium and bicarbonate reabsorption, insulin resistance, as the central feature of DM, could cause decreased urine pH ( Spatola *et al.*, 2018). Insulin resistance causes high levels of plasma-free fatty acids to reach the proximal tubule cells and interfere with the use of glutamine in ammonium production ( Bagnasco *et al.*, 1983; Lemieux *et al.*, 1977; Vinay *et al.*, 1976). Furthermore, insulin resistance may directly affect ammoniagenesis at the level of the kidney. This could lead to lower urinary PH which is a major risk factor for uric acid nephrolithiasis ( Asplin, 1996; Riese & Sakhaee, 1992). However, a lower level of urinary citrate also resulted in DM, which further induced hypercalciuria due to reduced citrate binding ( Spatola *et al.*, 2018). In addition, through isonatric inhibition of proximal tubular reabsorption of calcium, hyperglycemia and concurrent glycosuria acted separately to elevate urinary calcium excretion. These pathways, working together, facilitated the development of uric acid and calcium stone in diabetic patients ( Daudon *et al.*, 2006). Our results also provide evidence that there is a trend that the risk of developing nephrolithiasis was correlated with the higher the fasting plasma glucose. The links between distinct glycemic status and kidney stone disorder have been explored in recent research. Positive correlations between prediabetes and diabetes with the risk of nephrolithiasis were seen in the results and with insulin resistance, a greater risk of kidney stones was found ( Lien *et al.*, 2016; Weinberg *et al.*, 2014). Thus it is possible that kidney stones are developed after prediabetes, but before DM progresses. As a consequence of diagnostic chronology, this may partially explain the greater incidence of DM in kidney stone patients.

Insulin resistance may also play role in the link between obesity and nephrolithiasis which was reported in our research. In previous studies, urinary calcium excretion and body mass index (BMI) were found to be positively correlated ( Ekeruo *et al.*, 2004; Powell *et al.*, 2000; Siener *et al.*, 2004). It is well understood that one of the characteristics of obesity is insulin resistance. In patients with high BMI, it is found that increased uric acid excretion, low urinary pH both cause urinary supersaturation ( Maalouf *et al.*, 2004; Powell *et al.*, 2000). Insulin resistance with urinary pH <5.5 through the activation of exchanger NH3 in the proximal renal tubule thus reduces the synthesis and urinary excretion of ammonium ( Abate *et al.*, 2004; Chobanian & Hammerman, 1987; Klisic *et al.*, 2002). In acid urine, uric acid is found in an undissociated, readily precipitable state such that the deposition of uric acid stone can easily take place ( Pak *et al.*, 2001). Excess uric acid in the urine can also by a heterogeneous nucleation process, causing the precipitation of calcium oxalate dehydrate ( Coe *et al.*, 1980). The process of insulin resistance is not influenced by diet and lifestyle ( Kovesdy *et al.*, 2017).

Our study reveals that there was a 1.586 -fold increased risk of nephrolithiasis in participants with dyslipidemia. There has been previous meta-analysis that supports the correlation between dyslipidemia and nephrolithiasis ( Besiroglu & Ozbek, 2019). Our result was also supported by the fact that consuming statin medications can reduce stone genesis compared to those not consuming statin medications ( Inci *et al.*, 2012; Sur *et al.*, 2013). The study from Toricelli *et al.* ( Torricelli *et al.*, 2014) also reported low HDL and high TGs (triglycerides) are associated with lower urinary pH, and uric acid stones. Possible linking mechanism was explained that dyslipidemia and similar diseases are often concerned with systemic inflammation and oxidative stress ( Gorbachinsky *et al.*, 2010). They concluded that oxidative stress tends to be a key cytotoxic activity of the monohydrate calcium oxalate that can harm or destroy renal cells, and by unknown cellular physiological mechanisms could also contribute to stone forming. The oxidative stress cascade, which is the leading factor in cell damage, may be caused by dyslipidemia. Ultimately, the area of oxidatively stressed may lead to crystallization by the contribution of high calcium and phosphate or oxalate, and low urine citrate or magnesium. Moreover, lipid accumulation in the kidney which is defined as lipotoxicity caused changes in renal structure and function ( Gorbachinsky *et al.*, 2010). Lipotoxicity can induce an increased acid extraction, with decreased ammonia synthesis and ammonium excretion, resulting in a lower urinary pH. Bobulescu *et al.* ( Bobulescu *et al.*, 2008) in the rats study also revealed that TG steatosis around the tubular portions of the kidney is associated with low urinary pH and NH4 ^+^ and high titratable acidity. They demonstrated that the decrease in NH4+ excretion might be attributed to a lipotoxicity-induced reduction in NHE3 activity. In a follow up study of Bobulescu and colleagues, the reduction of renal steatosis in rats improved urinary NH4 ^+^, increased pH, reduced titratable acidity and finally increased citrate and brush border NHE3 levels and activity was demonstrated ( Bobulescu *et al.*, 2009).

The current findings have important public health implications in light of the current epidemics of obesity, diabetes, hypertension and dyslipidemia and suggest that these four factors play important roles in the development of nephrolithiasis. With this knowledge, determining common modifiable risk factors for the development of kidney stones might uncover new strategies enabling improved patient management and treatment of stone disease. Finally, there are several strengths of this review. First, the implementation of Bayesian framework in meta-analysis and meta regression could provide better confidence in the results; therefore, it gives clarity in the associations between nephrolithiasis and metabolic syndrome traits. Second, the results were tested using sensitivity analysis, trim-fill analysis, funnel plot, Begg’s rank correlation and Egger’s regression test to ensure that the results were relatively consistent and robust without the influence of confounders. Third, all studies selected for meta-analysis were of high quality, which report the association between the traits and nephrolithiasis. However, there are several limitations in this study. Firstly, the number of studies that can be pooled due to the fact that not many primary study have been conducted; secondly, not all studies included stone analysis therefore it is difficult to observe the association between metabolic syndrome components and different type of kidney stones; and thirdly, there are not many individual studies on metabolic syndrome and further updated meta-analyses need to be performed.

## Data availability

### Underlying data

All data underlying the results are available as part of the article and no additional source data are required.

### Extended data

Dryad: Data from: Association between metabolic syndrome components and the risk of developing nephrolithiasis: Bayesian meta-analysis and meta-regression with dose-response analysis, https://doi.org/10.5061/dryad.76hdr7svg ( Rahman *et al.*, 2021b).

This project contains the following extended data:

- Supplementary material 1. Funnel plot (a), trim-fill (b) and sensitivity analysis (c) of the association between metabolic syndrome and nephrolithiasis
- Supplementary material 2. Funnel plot (a), trim-fill (b) and sensitivity analysis (c) of the association between hypertension and nephrolithiasis
- Supplementary material 3. Funnel plot (a), trim-fill (b) and sensitivity analysis (c) of the association between DM and nephrolithiasis
- Supplementary material 4. Funnel plot (a), trim-fill (b) and sensitivity analysis (c) of the association between obesity and nephrolithiasis
- Supplementary material 5. Funnel plot (a), trim-fill (b) and sensitivity analysis (c) of the association between dyslipidemia and nephrolithiasis

Data are available under the terms of the Creative Commons Zero "No rights reserved" data waiver (CC0 1.0 Public domain dedication).

### Reporting guidelines

Figshare: PRISMA checklist for ‘Comparison of major bleeding in patients with acute coronary syndrome that underwent coronary artery bypass grafting treated with clopidogrel or ticagrelor: a systematic review and meta-analysis, https://doi.org/10.6084/m9.figshare.13585607 ( Rahman *et al.*, 2021a).

Data are available under the terms of the Creative Commons Attribution 4.0 International license (CC-BY 4.0).
