# Supplementary material for: Association between metabolic syndrome components and the risk of developing nephrolithiasis: A systematic review and bayesian meta-analysis
Source: F1000Res. 2021 Feb 11;10:104. [Version 1] doi: 10.12688/f1000research.28346.1 (PMC8577060; doi:10.12688/f1000research.28346.1)
Supplement: Peer review history

# Peer review: Reviewer response for version 1

**DOI:** 10.5256/f1000research.31355.r96149

**Published:** 2021-11-08

**Authors:** Muhammad Faruk

**Affiliations:** 1 Department of Surgery, Faculty of Medicine, Hasanuddin University, Makassar, Indonesia

This study assessed the association between metabolic syndrome and its components with the risk of developing nephrolithiasis by conducting systematic review, Bayesian random-effects meta-analysis, meta-regression and dose-response analysis. This study was done appropriately based on PRISMA flowchart. Risk of bias was also conducted of the included studies. This study has successfully presented the proper meta-analysis for this design. However, to complete this study for indexing, I personally recommended several revisions:

 1. Abstract:

- Introduction section: It is better to address meta-regression as the analysis to assess the correlation of association along with dose-response analysis
- Conclusion section: In reporting the association between predictors and nephrolithiasis, state only the predictors in which its coefficient was statistically significant

2. R language was not considered as a statistical software for data analysis. The software for data analysis should be written as "R" (Please refer to methods section in statistical analysis subsection).

 3. Please update the PRISMA flowchart (refer to PRISMA guideline 2009).

 4. Give the numbering of each Forrest plot in Figure 3 and numbering of each meta-regression plot in Figure 4. Design these figures so that it could be well presented.

 5. Uniformly decide the word choice of "traits" or "components", choose whether to use traits or components in the whole text, use one of these words consistently to avoid any misunderstanding.

 6. It is better to provide the meta-regression of hypertension in systolic blood pressure and diastolic blood pressure as it is important to explain the relationship to nephrolithiasis in differentiation for these two types of blood pressure.

 7. Meta-regression of body mass index  was sufficient in this study thus waist circumference meta-regression was not necessary to be included.

 8. Provide the value of coefficient and confidence interval of each meta-regression analysis in the result section so that better understanding of predictors-outcome relationship could be reached clearly.

Are the rationale for, and objectives of, the Systematic Review clearly stated?

Yes

Is the statistical analysis and its interpretation appropriate?

Yes

Are sufficient details of the methods and analysis provided to allow replication by others?

Yes

Are the conclusions drawn adequately supported by the results presented in the review?

Yes

Reviewer Expertise:

General Surgery, Urology

I confirm that I have read this submission and believe that I have an appropriate level of expertise to confirm that it is of an acceptable scientific standard.

# Peer review: Reviewer response for version 1

**DOI:** 10.5256/f1000research.31355.r80876

**Published:** 2021-03-22

**Authors:** Joseph philipraj, Vishnuvardhan Rudravaram

**Affiliations:** 1 Mahatma Gandhi Medical College and Research Institute, Pillayarkuppam, Puducherry, India; 2 Pondicherry university, Kalapet, Puducherry, India

This systematic review is appropriate for the journal with a global problem of Mets and Urolithiasis. The introduction part clearly explains the motivation. The manuscript is clear and balanced. The manuscript stays focused on the subject. Authors have gone through the process of searching relevant articles from all websites and of sufficient duration. The inclusion and exclusion criteria in the analysis have been clearly stated. The impact of the analysis is clearly stated.

 The statistical analysis supports the paper well. The interpretation of the results, visualisation are well presented. The tables and figures are clear, relevant and correct.

 The authors demonstrate the knowledge of basic composition skills, including word choice, sentence structure, paragraph development and grammar.

Limitations:

             The studies included in the meta-analysis have cross-sectional nature and hence ascertainment of temporal association is not possible which also dictates need for further prospective studies. The specific type of stone formation is not correlated with studies. Despite these limitations all studies included in the meta-analysis showed the same directionality in the association between urolithiasis and Mets.

Are the rationale for, and objectives of, the Systematic Review clearly stated?

Yes

Is the statistical analysis and its interpretation appropriate?

Yes

Are sufficient details of the methods and analysis provided to allow replication by others?

Yes

Are the conclusions drawn adequately supported by the results presented in the review?

Yes

Reviewer Expertise:

Endourogy, Urolithiasis, Andrology and Male Infertility, Uro-Oncology

We confirm that we have read this submission and believe that we have an appropriate level of expertise to confirm that it is of an acceptable scientific standard.
